# Suppression of LPS-Induced Inflammation by Chalcone Flavokawain A through Activation of Nrf2/ARE-Mediated Antioxidant Genes and Inhibition of ROS/NF*κ*B Signaling Pathways in Primary Splenocytes

**DOI:** 10.1155/2020/3476212

**Published:** 2020-06-12

**Authors:** Hsin-Ling Yang, Ting-Yu Yang, Yugandhar Vudhya Gowrisankar, Chun-Huei Liao, Jiunn-Wang Liao, Pei-Jane Huang, You-Cheng Hseu

**Affiliations:** ^1^Institute of Nutrition, College of Biopharmaceutical and Food Sciences, China Medical University, Taichung 40402, Taiwan; ^2^Department of Cosmeceutics, College of Biopharmaceutical and Food Sciences, China Medical University, Taichung 40402, Taiwan; ^3^Graduate Institute of Veterinary Pathology, National Chung-Hsing University, Taichung 402, Taiwan; ^4^Department of Health and Nutrition Biotechnology, Asia University, Taichung 41354, Taiwan; ^5^Chinese Medicine Research Center, China Medical University, Taichung 40402, Taiwan; ^6^Research Center of Chinese Herbal Medicine, China Medical University, Taichung 40402, Taiwan

## Abstract

Oxidative stress is an important contributing factor for inflammation. *Piper methysticum*, also known as *Kava*-*kava*, is a shrub whose root extract has been consumed as a drink by the pacific islanders for a long time. Flavokawain A (FKA) is a novel chalcone derived from the *kava* plant that is known to have medicinal properties. This study was aimed at demonstrating the antioxidant molecular mechanisms mediated by FKA on lipopolysaccharide- (LPS-) induced inflammation in BALB/c mouse-derived primary splenocytes. *In vitro* data show that the nontoxic concentrations of FKA (2-30 *μ*M) significantly suppressed the proinflammatory cytokine (TNF-*α*, IL-1*β*, and IL-6) release but induced the secretion of interleukin-10 (IL-10), an anti-inflammatory cytokine. It was also shown that FKA pretreatment significantly downregulated the LPS-induced ROS production and blocked the activation of the NF*κ*B (p65) pathway leading to the significant suppression of iNOS, COX-2, TNF-*α*, and IL-1*β* protein expressions. Notably, FKA favored the nuclear translocation of Nrf2 leading to the downstream expression of antioxidant proteins HO-1, NQO-1, and *γ*-GCLC via the Nrf2/ARE signaling pathway signifying the FKA's potent antioxidant mechanism in these cells. Supporting the *in vitro* data, the *ex vivo* data obtained from primary splenocytes derived from the FKA-preadministered BALB/c mice (orally) show that FKA significantly suppressed the proinflammatory cytokine (TNF-*α*, IL-1*β*, and IL-6) secretion in control-, LPS-, or Concanavalin A- (Con A-) stimulated cells. A significant decrease in the ratios of pro- and anti-inflammatory cytokines (IL-6/IL-10; TNF-*α*/IL-10) showed that FKA possesses strong anti-inflammatory properties. Furthermore, BALB/c mice induced with experimental pancreatitis using cholecystokinin- (CCK-) 8 showed decreased serum lipase levels due to FKA pretreatment. We conclude that with its potent antioxidant and anti-inflammatory properties, chalcone flavokawain A could be a novel therapeutic agent in the treatment of inflammation-associated diseases.

## 1. Introduction

Inflammation is characterized as a protective biological response with complicated mechanisms and implicates immune cells and molecular mediators secreted from the cells that act against pathogens, damaged cells, or other irritants. The inflammation process rules out the initial causes of cell injury, cleans away necrotic cells, and begins tissue repairs [1]. There is a dynamic and ever-shifting balance exits between pro- and anti-inflammatory components of the immune system [2]. An uncontrolled shift of this balance towards excessive production of proinflammatory cytokines causes several major cellular events that lead to the pathogenesis and progression of inflammatory responses. Splenocytes are a type of white blood cells from the splenic origin that consists of a variety of T and B lymphocytes, dendritic cells, and macrophages which have different immune functions and release various factors in response to inflammatory and anti-inflammatory agents [3]. Various transcription factors and cellular signaling pathways are involved in the expression of proinflammatory genes in macrophages [4].

LPS (the main component of gram-negative bacterial endotoxin) is one of the primary causes of sepsis. The administration of LPS in laboratory animals duplicates that of an experimental inflammatory response. During an inflammation process, the MAPKs p38, JNK, and ERK are involved in the expression of proinflammatory genes. NF*κ*B is a crucial factor and plays a major role in the regulation of gene expression patterns of various genes in both innate and adaptive immunities. Upon stimulation with LPS, the activated MAPKs mediate the signaling cascades leading to the activation of NF*κ*B in activated macrophages [4, 5]. Further, LPS-mediated splenocyte activation can initiate oxygen uptake to drive oxidative stress-induced inflammation in the immune cells leading to ROS production [6]. Mulder et al. suggested that in response to activation with interferon-*γ* (IFN-*γ*) and lipopolysaccharide (LPS), spleen-derived macrophages readily acquired a proinflammatory status (M1 type) indicated by the upregulation of nitric oxide (NO) production, prostaglandin E2 (PEG2), tumor necrosis factor-*α* (TNF-*α*), and interleukins through NF*κ*B activation [7, 8]. These proinflammatory molecules participate in the development of inflammatory reactions [9, 10].

The expression levels of cytoprotective enzymes, which are a response to oxidative stress, are primarily regulated at the transcriptional level. The Nrf2/ARE pathway controls a network of cytoprotective genes that defend against the damaging effects of oxidative and electrophilic stress and inflammation [11]. Nrf2 is an important protein that participates in the coordination of transcriptional induction for various antioxidant enzymes, which also protects them from LPS-stimulated inflammation [12]. Nrf2 is a member of the basic leucine zipper (bZIP) family of transcriptional activator proteins, and it is activated by endogenous products of oxidative stress. Nrf2 is bound to Keap-1 and stays in the cytoplasm. Due to the ‘inducers,' Nrf2-Keap1 complex disrupts and Nrf2 undergoes rapid translocation to the nucleus, binds to antioxidant response elements (ARE), and induces the expression of antioxidant and phase II enzymes, such as heme oxygenase-1 (HO-1), NAD(P)H-quinone oxidoreductase-1 (NQO-1), gamma-glutamylcysteine synthetase (*γ*-GCLC), glutathione (GSH), and glutathione S-transferase A2 (GST-A2) [13]. This group of enzymes has cytoprotective, antioxidant, and anti-inflammatory effects in endotoxin-induced macrophages. Several new and natural compounds and crude herbal extracts of medicinal herbs induced the expression of phase II detoxifying enzymes in different cell types has been reported. A few of these reports, Nrf2-mediated genes employ antioxidant and anti-inflammatory activities [14]. Therefore, an effective strategy would be to reverse the negative effects of LPS-induced ROS generation through the exogenous supplementation of antioxidants.

Kava-kava, scientifically known as *Piper methysticum,* is a shrub belonging to the pepper family *Piperaceae*. It is widely cultivated in various parts of the world, mainly in the Pacific islands. The aqueous root extract of kava has been consumed as a drink that has a strong odor and a pungent taste with sedative, anesthetic, and euphoriant properties. Kavalactones and chalcones are two major phytochemical ingredients present in this plant. The chalcones are derived from flavonoids with a basic molecular structure of two aromatic rings linked by an unsaturated three-carbon bridge. Chalcones in the kava plant can be recognized by their yellow appearance and are named flavokawains. Flavokawain A (FKA) is the major constituent of chalcones (0.46%) derived from kava extracts [15]. Splenocytes consist of a variety of cell populations such as T and B lymphocytes, monocytes, and macrophages, which have different immune functions and release different factors in response to the inflammatory and anti-inflammatory agents. A previous study indicated that FKA suppresses iNOS and COX-2 expression via blockade of NF*κ*B and AP-1 activation in RAW 264.7 macrophages [16]. However, little is known about the anti-inflammatory activity of FKA in splenocytes. In this study, we further demonstrated the molecular signaling pathways associated with FKA-mediated antioxidant and anti-inflammatory properties in primary splenocytes isolated from the BALB/c mice that were challenged to the LPS-induced inflammation.

## 2. Materials and Methods

### 2.1. Reagents and Antibodies

This study bought Roswell Park Memorial Institute (RPMI)-1640, fetal bovine serum (FBS), Dulbecco's Modified Eagle's medium (DMEM), and penicillin/streptomycin/amphotericin from Gibco BRL/Invitrogen (Carlsbad, CA, USA). We obtained LPS (from *Escherichia coli* 055: B5), 3-(4,5-dimethylthiazol-2-yl)-2,5-diphenyltetrazolium bromide (MTT), and 2′,7′-dihydrofluorescein-diacetate (DCFH_2_-DA) from Sigma-Aldrich (St. Louis, MO, USA). We purchased flavokawain A (FKA, purity≧98%) from LKT Laboratories, Inc. (St. Paul, MN, USA). We obtained the antibodies for p65 NF*κ*B, histone H3from Cell Signaling Technology Inc. (Danvers, MA). We purchased antibodies against iNOS, COX-2, Nrf2, NQO-1, and *β*-actin from Santa Cruz (Heidelberg, Germany). We procured mouse monoclonal antibodies against TNF-*α*, and IL-1*β* from Abcam (Cambridge, UK). We obtained anti-*γ*-GCLC and HO-1 antibodies from Gene Tex Inc. (San Antonio, TX, USA). We obtained all other chemicals and general lab equipment from either Merck & Co., Inc. (Darmstadt, Germany) or Sigma-Aldrich (St. Louis, MO, USA).

### 2.2. Laboratory Animals

We purchased 6–8-week-old female BALB/c mice (20-25 g) from the National Animal Center (Taipei, Taiwan). They were maintained in a pathogen-free set up with a 12-h/12-h light/dull cycle. There was free access to water and rat chow (Oriental Yeast Co. Ltd., Tokyo, Japan) for all mice [17]. In accordance with “The Guidelines for the Care and Use of Laboratory Animals” suggested by the Chinese Society of Animal Science, Taiwan, all animal experiments in this study were performed as indicated. The animal conventions were endorsed by the Institutional Animal Care and Use Committee (IACUC) of China Medical University.

### 2.3. Primary Splenocyte Preparation

Primary splenocytes were aseptically isolated from the 8–12-week-old female BALB/c mice and kept in the RPMI-1640 medium. This medium was supplemented with 0.1% of the penicillin-streptomycin-amphotericin solution and 2% of 50x tissue culture medium (TCM, a serum substitution purchased from Protide Pharmaceuticals, Inc., Lake Zurich, IL) [18]. Suitable cell numbers were evaluated using the trypan blue exclusion method and a hemocytometer. Segregated splenocytes from every animal were acclimated to a cell density of 1 × 10^7^ cells/mL of the RPMI-1640 medium. We procured RAW264.7 (the murine macrophage cell line) from the American Type Culture Collection (ATCC, Rockville, MD, USA). It was then cultured in 1% penicillin-streptomycin, DMEM containing 2 mM glutamine, and 10% heat-inactivated FBS at 37°C in a humidified atmosphere with 5% CO_2_.

### 2.4. *In Vitro* Stimulation Assays

The primary splenocyte or RAW264.7 macrophage cultures were pretreated with FKA for 2 h. After incubation, PBS washed cells were exposed to the fresh medium supplemented with or without LPS (prepared in pH 7.2 PBS). Liu et al. reported that higher concentrations of (5–20 *μ*g/mL) LPS cause significant cytotoxicity to the cells [19]. Also, Kwon et al. demonstrated the inflammation studies in RAW267.4 macrophages by using 1 *μ*g/mL of LPS [16]. Based on this 2.5 *μ*g/mL and 1 *μ*g/mL concentrations of LPS were used for splenocytes and RAW264.7 cells, respectively, in the entire study.

For the measurement of TNF-*α*, IL-1*β*, IL-2, IL-6, or IL-10 levels in the cell culture medium, approximately 6.5 × 10^5^ cells/well of splenocytes were cultured in a 12-well plate. These cells were either treated with FKA (0-30 *μ*M, 72 h) to measure the effect of FKA on cytokine secretion (or) pretreated with FKA (0-30 *μ*M, 2 h) followed by LPS (2.5 *μ*g/mL, 72 h) to measure the protective effect of FKA on cytokine secretion from LPS-stimulated cells using the ELISA method. The cytokines, TNF-*α*, IL-1*β*, IL-2, IL-6, or IL-10 were quantified by their respective ELISA kits (R&D Systems, Minneapolis, MN, USA) using the manufacturer's protocols [20].

### 2.5. MTT Assay

MTT colorimetric assay measured the effect of FKA on cell viability [21]. Splenocytes (1 × 10^7^ cells/mL in 96-well plate) or macrophages (4 × 10^5^ cells/mL in 12-well plate) were pretreated with FKA for 2 h, followed by treatment with or without LPS (2.5 or 1 *μ*g/mL) for 72 h. After treatment, MTT (0.5 mg/mL) was added to each well and incubated at 37°C for 4 h. After the incubation period, using the 400 *μ*L DMSO, MTT formazan crystals were dissolved and the absorbance (A_570_) was measured through an ELISA microplate reader (*μ*-Quant, Winooski, VT, USA). The cell viability was expressed as a percentage using the following formula: (treated cells/untreated cells) × 100.

### 2.6. Measurement of Intracellular ROS Accumulation

The intracellular ROS accumulation was measured by the DCFH_2_-DA fluorescence dye method [22]. Briefly, 1 × 10^7^ cells/mL were seeded in a 6-well plate (primary splenocytes) or 4 × 10^5^ cells/mL were seeded in a 12-well plate (RAW 264.7 macrophages) and then pretreated with different concentrations of FKA (for 2 h) followed by treatment with or without LPS. After incubation, 10 *μ*M of DCFH_2_-DA was added to the culture medium and the incubation was continued for 30 min at 37°C. The intracellular ROS, as indicated by the dichlorofluorescein (DCF) fluorescence intensity, was measured using a Becton-Dickinson FACSCalibur flow cytometer for suspended primary splenocytes (Becton Dickinson, NJ) or fluorescence microscopy for adherent RAW 264.7 macrophages (200x magnification, Olympus, Center Valley, PA, USA). The ROS levels were measured by comparing the exhibited fluorescence intensity from any treated cells compared to the vehicle-treated cells and were assigned to 1-fold arbitrarily.

### 2.7. Preparation of Cell Extracts and Western Blot Analysis

Approximately 1 × 10^7^ cells/mL of primary splenocytes (seeded in 60 mm dish) or 4 × 10^6^ cells/mL of RAW264.7 macrophages (seeded in a 100 mm dish) pretreated with different concentrations of FKA (2.5–30 *μ*M) for 2 h followed by exposing to LPS (2.5 or 1 *μ*g/mL) at different time points (1–24 h). The untreated cells were the control cells. After treatment, the cytoplasmic, nuclear fractions of the proteins were harvested using protein extracting reagents (Pierce Biotechnology, Rockford, IL, USA). The concentrations of extracted protein fractions were measured using the BCA protein assay method (Bio-Rad, Hercules, CA, USA). Equal quantities of denatured proteins (50 *μ*g) were resolved on 8–15% gradient SDS-PAGE, followed by transfer on to PVDF membranes. Blocking buffer was used to block these (5% nonfat dry milk for 30 min) and then incubation with various primary antibodies overnight. On the next day, membranes were washed and incubated with secondary antibodies for 2 h. After the incubation, PBS-washed membranes were developed and the protein bands were visualized using a chemiluminescence substrate (Pierce Biotechnology, Rockford, IL, USA). Using AlphaEase (Genetic Technology Inc. Miami, FL, USA) densitometric analysis was carried out and the protein expression data were represented as fold over control. Throughout the western blot experiments, either *β*-actin (for cytosolic proteins) or histone (for nuclear proteins) proteins were considered internal protein controls [23].

### 2.8. *In Vivo* Demonstration of Pro- and Anti-inflammatory Cytokine Levels in Control-, LPS-, or Con A-Stimulated Splenocytes Isolated from the FKA-Administered BALB/c Mice

Eight BALB/c mice were randomly segregated into two groups of four mice each. These mice were orally administered with 0 (control) or 30 mg/kg of FKA (solubilized in 0.1% DMSO) for 4 h. At the end of the incubation period, all mice were sacrificed and primary splenocytes were aseptically isolated as per the procedure described above [18]. Primary splenocytes were seeded in 12-well plates (1 × 10^7^ cells/mL) and were stimulated with control (saline), LPS (2.5 *μ*g/mL), or concanavalin A (Con A, 2.5 *μ*g/mL) for 72 h. After the incubation period, the concentrations of secreted TNF-*α*, IL-1*β*, IL-2, IL-6, or IL-10 levels in the control-, LPS-, or Con A- stimulated cell culture medium were measured using the ELISA kits (R&D Systems, Minneapolis, MN, USA) in accordance with the manufacturer's protocol.

### 2.9. Determination of Serum Lipase Concentrations in Cholecystokinin-8- (CCK-8-) Induced Experimental Pancreatitis of BALB/c Mice

Twenty BALB/c mice were randomly placed into five different groups and each group was made up of four mice. The mice were administered either oral administration of FKA (in 0.1% DMSO) and/or intraperitoneal (IP) injections of cholecystokinin-8 (CCK-8). The treatments were as follows: (a) vehicle (0.1% DMSO), (b) 100 *μ*g/kg of CCK-8 alone, (c) 15 mg/kg of FKA+100 *μ*g/kg of CCK-8, (d) 30 mg/kg of FKA+100 *μ*g/kg of CCK-8, and (e) 30 mg/kg of FKA alone. In the case of groups ‘c' and ‘d,' CCK-8 was injected into the mice for 0.5, 1.5, 2.5, and 4 h after the oral administration of FKA [22]. After the CCK-8 injections, the blood samples for the mice were collected from the retroorbital sinus and the serum lipase levels were measured by using a commercial serum lipase ELISA kit (R&D System, Minneapolis, MN, USA).

### 2.10. Statistical Analyses

In this study, data were represented as mean ± standard deviation (mean ± SD) of three or more independent experiments. All data were analyzed using analysis of variance (ANOVA), followed by Dunnett's test for pairwise comparison. Statistical significance was assigned as ^∗^*p* < 0.05, ^∗∗^*p* < 0.01, and ^∗∗∗^*p* < 0.001 compared to the untreated control cells and ^#^*p* < 0.05,^##^*p* < 0.01, and ^###^*p* < 0.001 compared to the LPS-treated group.

## 3. Results

### 3.1. FKA Mediated Differential Secretion Patterns of Pro- and Anti-inflammatory Cytokines in Primary Splenocytes

**Figure 1:**
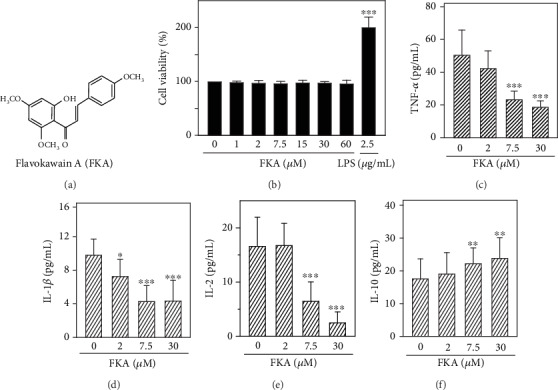
FKA attenuated the secretions of proinflammatory TNF-*α*, IL-1*β*, and IL-2 levels but enhanced the anti-inflammatory IL-10 levels in primary splenocyte cell culture medium. (a) Chemical structure of flavokawain A (FKA). (b) Effect of FKA on cell viability. Cells were treated with FKA (1-60 *μ*M) or LPS (2.5 *μ*g/mL) for 72 h. Cell viability was determined by MTT assay. (c–f) Splenocytes were pretreated with 0, 2, 7.5, and 30 *μ*M of FKA for 72 h. After the incubation period, the levels of pro- and anti-inflammatory cytokines in the cell culture medium were measured by commercial ELISA kits. The data were represented as the mean ± SD of six experiments. Statistical significance was assigned as ^∗^*p* < 0.05, ^∗∗^*p* < 0.01, and ^∗∗∗^*p* < 0.001 compared to the control cells.

We first determined the subtoxic dosage of FKA (Figure 1(a)) on primary splenocytes derived from the BALB/c mice. Primary splenocytes were treated with increasing concentrations of FKA (1 to 60 *μ*M) or 2.5 *μ*g/mL of LPS for 72 h. After incubation has concluded, an MTT assay was conducted to determine cell viability. The data showed that compared with control cells, splenocytes exposed to FKA did not enhance the proliferative state of splenocytes and did not induce any significant toxicity as well [24]. Based on these observations, 30 *μ*M FKA has been used as the maximum concentration in the entire study (Figure 1(b)). Later, we tested the effect of FKA concentration on the secretion patterns of pro- and anti-inflammatory cytokines in the FKA-stimulated primary splenocyte cell culture medium. ELISA data showed that FKA dose dependently and significantly suppressed the release of proinflammatory TNF-*α*, IL-1*β*, and IL-2 but upregulated the release of anti-inflammatory IL-10 from primary splenocytes (Figures 1(c)–1(f)). This data suggested that FKA showed anti-inflammatory properties in primary splenocytes.

### 3.2. FKA Suppressed the LPS-Induced Proinflammatory Cytokine Secretion in Primary Splenocytes

**Figure 2:**
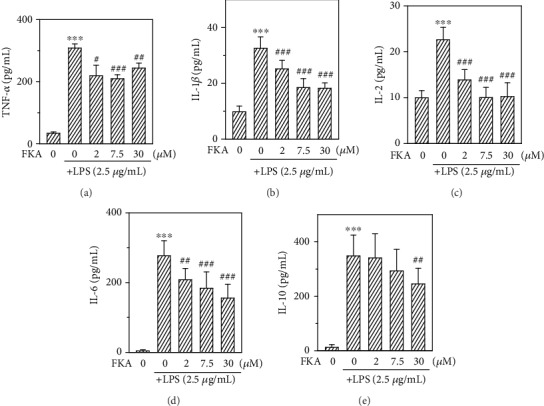
FKA suppressed the LPS-induced TNF-*α*, IL-1*β*, IL-2, and IL-6 secretions in primary splenocytes. Splenocytes were pretreated with FKA (0, 2-30 *μ*M) for 2 h, followed by LPS (2.5 *μ*g/mL) stimulation for 72 h. The secretions of TNF-*α* (a), IL-1*β* (b), IL-2 (c), IL-6 (d), and IL-10 (e) were measured through commercial ELISA kits. The data were represented as the mean ± SD of three experiments. Statistical significance was assigned as ^∗∗∗^*p* < 0.001 compared to the control cells and ^#^*p* < 0.05, ^##^*p* < 0.01, and ^###^*p* < 0.001 compared to the LPS-treated cells.

The anti-inflammatory efficacy of FKA was demonstrated in LPS-stimulated primary splenocytes. Cells were pretreated with different concentrations (0, 2–30 *μ*M) of FKA for 2 h followed by LPS stimulation (2.5 *μ*g/mL) for 72 h.  Figure 2 shows that, in comparison with control cells, LPS alone stimulation has significantly enhanced the productions of both TNF-*α*, IL-1*β*, IL-2, and IL-6 and IL-10 cytokines. However, FKA pretreatment dose dependently and significantly suppressed the release of proinflammatory cytokines (TNF-*α*, IL-1*β*, IL-2, and IL-6) signifying the protective role of FKA in LPS-induced inflammation in primary splenocytes. Interestingly, FKA has a negligible effect on LPS-stimulated anti-inflammatory IL-10 secretion.

### 3.3. FKA Dose Dependently Suppressed the Intracellular ROS-Mediated NF*κ*B Activation and Its Associated Inflammatory Cytokines in LPS-Stimulated Primary Splenocytes

**Figure 3:**
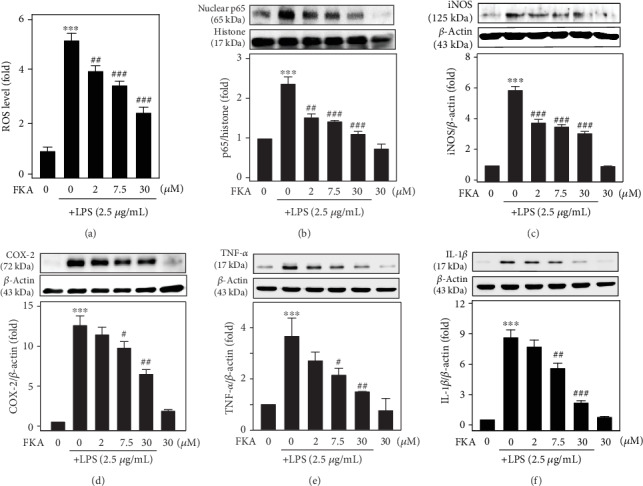
LPS-stimulated ROS levels, NF*κ*B activation, and iNOS, COX-2, TNF-*α*, and IL-1*β* protein expressions were suppressed in FKA-pretreated primary splenocytes. (a) Primary splenocytes were pretreated with FKA (0, 2-30 *μ*M for 2 h) followed by stimulation with LPS (2.5 *μ*g/mL) for 18 h. The intracellular ROS levels were measured by the DCF fluorescence technique using the flow cytometry method. (b) Western blotting showing the changes in NF*κ*B (p65). Cells were pretreated with FKA (2.5-30 *μ*M) for 2 h, followed by LPS stimulation (2.5 *μ*g/mL) for 1 h. (c–f) Western blotting showing the changes in iNOS (c), COX-2 (d), TNF-*α* (e), and IL-1*β* (f) levels. Cells were pretreated with FKA (0, 2-30 *μ*M, 2 h) followed by LPS stimulation (2.5 *μ*g/mL, 18 h). Equal concentrations of protein samples (50 *μ*g) were resolved on 8-15% SDS-PAGE. The results obtained were mean ± SD of three experiments. Statistical significance was assigned as ^∗^*p* < 0.05, ^∗∗^*p* < 0.01, and ^∗∗∗^*p* < 0.001 compared to the control cells and ^#^*p* < 0.05, ^##^*p* < 0.01, and ^###^*p* < 0.001 compared to the LPS-treated cells.

ROS plays an important role in the modulation of various inflammatory mediators [25]. The generation of intracellular ROS was measured using the DCFH_2_-DA fluorescence method. Splenocytes were pretreated with different concentrations of FKA (0, 2–30 *μ*M for 2 h) followed by LPS stimulation (2.5 *μ*g/mL) for 18 h. These cells were used to measure the accumulation of intracellular ROS levels. Flow cytometry data indicated that FKA pretreatment dose dependently and significantly attenuated the LPS-induced ROS accumulation (Figure 3(a)).

Multiple aspects of innate and adaptive immune functions are regulated by the transcription factor NF*κ*B. It also serves as an important mediator of an inflammatory response [7]. We also tested the role of NF*κ*B in the suppression of inflammation in FKA-pretreated and LPS-stimulated splenocytes. Western blot data obtained from the splenocyte nuclear protein extracts showed that LPS (2.5 *μ*g/mL for 1 h) stimulation induced a remarkable increase in the p65 levels. Similar to the ROS data, FKA pretreatment significantly attenuated the LPS-induced p65 levels at all the concentrations of FKA (Figure 3(b)).

Later, we tested the effect of FKA pretreatment on the expression patterns of iNOS and COX-2 enzymes and TNF-*α* and IL-1*β* (proinflammatory cytokines). Western blot data showed that 2.5 *μ*g/mL LPS stimulation (for 18 h) overexpressed the expressions of iNOS, COX-2, TNF-*α*, and IL-1*β*. However, this effect was significantly and remarkably suppressed in the present higher concentrations of FKA (>7.5 *μ*M) suggesting that FKA suppressed the transcriptional activation of p65 leading to further suppression of inflammatory enzymes and cytokine expressions (Figures 3(c)–3(f)).

### 3.4. FKA Mediated Antioxidant Gene Expression via Nrf2 Pathway in Primary Splenocytes

**Figure 4:**
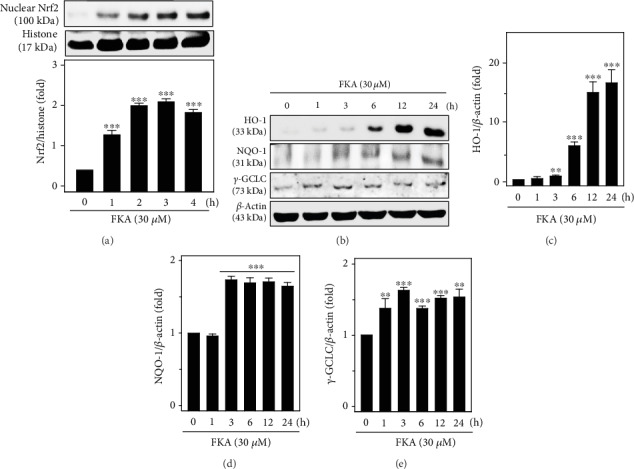
FKA induced HO-1, NQO-1, and *γ*-GCLC antioxidant gene expression via the Nrf2 signaling pathway in primary splenocytes. (a) Nuclear translocation of Nrf2 was mediated by FKA. Cells were treated with 30 *μ*M FKA for 1-4 h. Using the western blot technique, the expression of nuclear Nrf2 levels was measured against the internal control protein histone. (b–e) FKA mediated the upregulation of HO-1, NQO-1, and *γ*-GCLC proteins. Cells were treated with 30 *μ*M FKA for 1-24 h. The expressions of HO-1, NQO-1, and *γ*-GCLC proteins were determined by western blot method and were represented as fold change over the untreated control splenocytes. *β*-Actin acts as an internal protein control. Results were calculated as fold change over the control splenocytes that were arbitrarily assigned the value of 100%. Data were represented as mean ± SD of three experiments. Statistical significance was assigned as ^∗∗^*p* < 0.01; ^∗∗∗^*p* < 0.001 compared to the control cells.

Previous studies have shown that Nrf2 is a part of the anti-inflammatory process because it mediates the recruitment of inflammatory cells and regulates gene expression via the antioxidant response element (ARE). Anti-inflammatory gene expression is primarily regulated by the Keap-1/Nrf2/ARE signaling pathway. It also inhibits the progression of inflammation [26]. To test this, primary splenocytes were treated with 30 *μ*M FKA for 1–4 h and the expression of nuclear translocation of Nrf2 protein was measured via the western blot method. Data showed that FKA time dependently induced the nuclear translocation of Nrf2 with a significant increase in the expression of Nrf2 at 3 h time point (Figure 4(a)). The data shows that FKA is crucial in the activation and nuclear translocation of Nrf2 in splenocytes. Further, we demonstrated the effect of time on the FKA-mediated downstream expressions of HO-1, NQO-1, and *γ*-GCLC antioxidant proteins. Primary splenocytes were treated with 30 *μ*M FKA for 1–24 h, and the protein expression of various antioxidant proteins was measured. The western blot data in this study showed that FKA treatment time dependently upregulated the expressions of the HO-1, NQO-1, and *γ*-GCLC proteins with the maximum expressions observed at longer time points (>6 h) (Figure 4(b)). The data were calculated as the fold-over basal levels of antioxidant protein expression at different time points that were normalized with the *β*-actin internal control and represented as the ratio value. Data showed that FKA has a differential but significant effect on the expression patterns of HO-1, NQO-1, and *γ*-GCLC antioxidant proteins (Figures 4(c)–4(e)).

### 3.5. FKA Induced ROS Production in Primary Splenocytes

**Figure 5:**
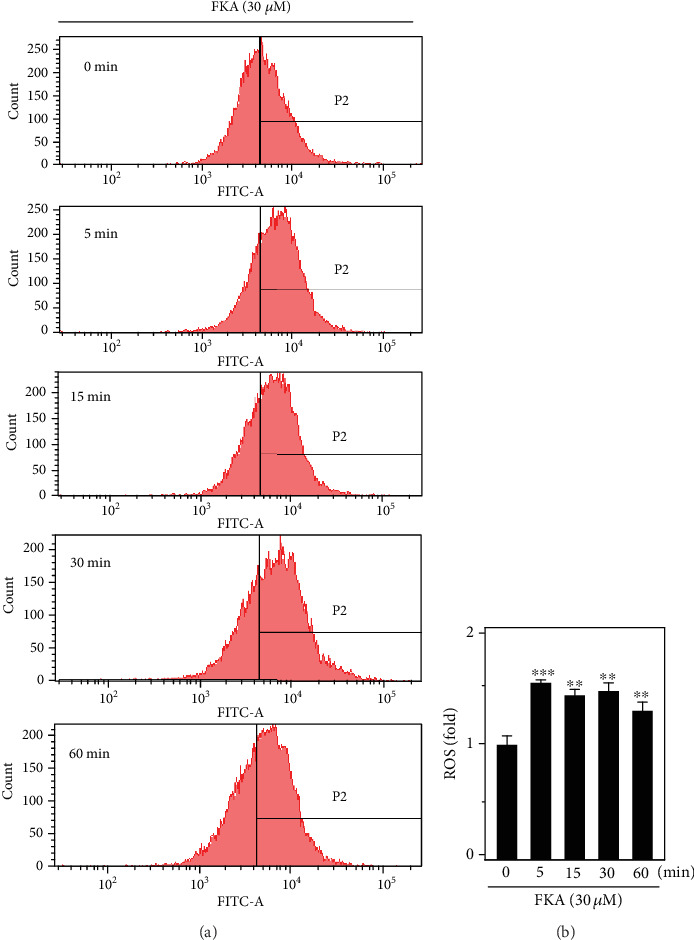
FKA induced ROS generation in primary splenocytes. (a) Splenocytes were pretreated with FKA (30 *μ*M) for 0-60 min. The generation of intracellular ROS levels was indicated by the DCF fluorescence method that was measured by flow cytometry. (b) Data were presented as fold change of ROS over the FKA untreated control cells. Results were represented as the mean ± SD of three experiments. Statistical significance was assigned as ^∗^*p* < 0.05, ^∗∗^*p* < 0.01, and ^∗∗∗^*p* < 0.001 compared to the control cells.

Nrf2 activation regulates several signaling cascades, including ROS. They are formed as a natural byproduct of the normal metabolism of oxygen and have important roles in cell signaling and homeostasis. Although ROS are harmful, low concentrations of ROS are believed to be involved in redox signaling that may contribute to normal cellular functions, adaptation, and disease prevention [27]. In this study, we tested the effect of FKA-mediated ROS generation in primary splenocytes. For this, cells were treated with FKA (30 *μ*M) for 0-60 min and the generation of intracellular ROS levels were measured by the DCF fluorescence method. Our flow cytometry data indicated that compared to the untreated control cells, FKA significantly upregulated (approximately 1.5-fold) the induction of ROS within 5 min after exposure to FKA in primary splenocytes and this pattern was continued up to 60 min (Figures 5(a) and 5(b)).

### 3.6. Effect of Pro- and Anti-inflammatory Cytokine Expression in LPS- or Con A-Stimulated Primary Splenocytes Isolated from the *In Vivo* FKA Pretreatment to the BALB/c Mice

Primary splenocytes were isolated from the BALB/c mice that were administered with FKA. These cells were stimulated with saline (control), LPS (2.5 *μ*g/mL), or Con A (2.5 *μ*g/mL) for 72 h, and the effect of *in vivo* FKA pretreatment on the expression patterns of TNF-*α*, IL-1*β*, IL-2, and IL-6 and IL-10 were measured by the secretion of related cytokines in a cell culture medium using an ELISA kit for each cytokine. Our data showed that FKA pretreatment significantly downregulated the production of TNF-*α*, IL-1*β*, IL-2, and IL-6 in the control-, LPS-, or Con A-stimulated splenocytes. In contrast to this, IL-10 levels were upregulated in the control- or Con A-stimulated splenocytes. But, this upregulation was not statistically significant. Interestingly, the TNF-*α*/IL-10 and IL-6/IL-10 ratio values were decreased in the FKA-treated group which was statistically significant. All this data suggested that FKA pretreatment plays an anti-inflammatory role in primary splenocytes (Table 1).

### 3.7. FKA Attenuated Serum Lipase Levels in BALB/c Mice Induced with Experimental Pancreatitis

**Figure 6:**
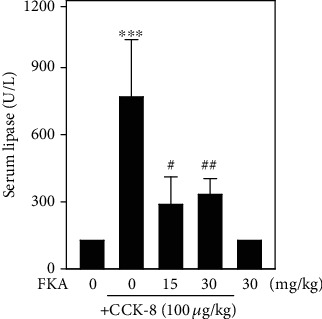
FKA suppressed the serum lipase levels in CCK-8-induced experimental pancreatitis in BALB/c mice. BALB/c mice were orally administered with FKA (15 or 30 mg/kg in 0.1% DMSO for 2 h) followed by intraperitoneal (i.p.) injections of cholecystokinin-8 (CCK-8). 0.5, 1.5, 2.5, and 4 h after the CCK-8 injections, blood samples were collected from the retroorbital sinus of all mice and serum lipase levels were measured using a commercial serum lipase ELISA kit (R&D System, Minneapolis, MN, USA). Results were represented as the mean ± SD of four experiments. Significant at ^∗∗^*p* < 0.01 compared to the control animals; significant at ^#^*p* < 0.05 compared to the CCK8- administered animals.

Seifert et al. have suggested that serum lipase levels are an important marker characterized by an upregulation that can be observed in animals who were given pancreatitis [28]. In this study, BALB/c mice were orally administered with FKA (15 or 30 mg/kg in 0.1% DMSO) for 2 h, which was then followed by intraperitoneal (i.p.) injections of cholecystokinin-8 (CCK-8) for 0.5, 1.5, 2.5, and 4 h. After treatment, blood samples were collected from all mice and the serum lipase levels were estimated using the ELISA kits. Our results showed that mice injected with CCK-8 alone showed significantly elevated serum lipase levels. This effect was significantly attenuated by FKA. The data clearly showed the anti-inflammatory properties of FKA (Figure 6).

### 3.8. FKA Attenuates LPS-Stimulated ROS Generation through the Nrf2/ARE Signaling Pathways in Murine RAW 264.7 Macrophages

**Figure 7:**
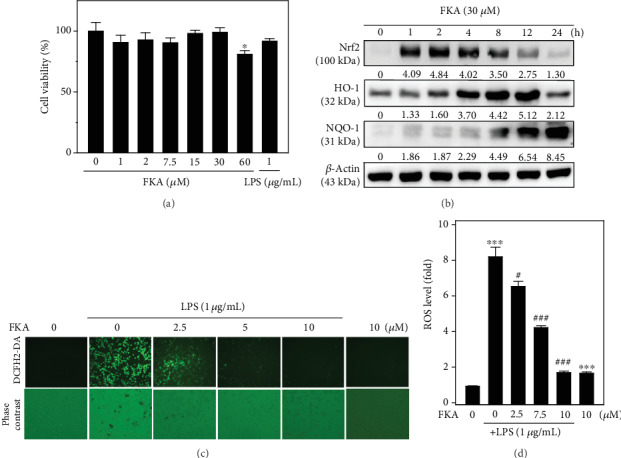
FKA attenuated the LPS-stimulated ROS generation in RAW 264.7 macrophages through the Nrf2 pathway. (a) Effect of FKA concentration on the viability of RAW 264.7 macrophages. Cells were treated with FKA (1-60 *μ*M) or LPS (1 *μ*g/mL) for 24 h. Cell viability was determined by MTT assay. (b) FKA induced the expression of Nrf2, HO-1, and NQO-1 proteins in RAW 264.7 macrophages. Cells were pretreated with 30 *μ*M FKA for 1-24 h. The protein expressions of Nrf2, HO-1, and NQO-1 were determined by the western blot technique. *β*-Actin was used as internal controls. (c, d) FKA attenuates ROS generation in LPS-stimulated RAW 264.7 macrophages. Cells were pretreated with FKA (0, 2.5-10 *μ*M) for 2 h and then stimulated without or with LPS (1 *μ*g/mL) for 24 h. The intracellular ROS levels were measured by the DCF fluorescence method using fluorescence microscopy (200x magnification). Data were represented as fold change along with the fluorescent image. Results were represented as the mean ± SD of three experiments. Statistical significance was assigned as ^∗^*p* < 0.05, ^∗∗^*p* < 0.01, and ^∗∗∗^*p* < 0.001 compared to the control cells and ^#^*p* < 0.05, ^##^*p* < 0.01, and ^###^*p* < 0.001 compared to the LPS-treated cells.

FKA suppressed the expression of iNOS and COX-2 and then the production of NO and PGE2 in the LPS-stimulated murine RAW264.7 macrophages [16]. Further, FKA inhibited the activation of the NF*κ*B and AP-1 signaling pathways [16]. Therefore, RAW 264.7 cells were used to investigate the FKA-activated Nrf2/ARE signaling pathways as involved in the suppression of LPS-induced inflammation. We first tested the effect of FKA on the viability of RAW 264.7 cells. These macrophages were exposed to increasing concentrations of FKA (0, 1–60 *μ*M) for 2 h and then stimulated without or with LPS (1 *μ*g/mL) for 24 h. After treatments, an MTT assay was performed to measure its viability. Data showed that up to 30 *μ*M concentrations, FKA was nontoxic to macrophages (i.e., no significant reductions in macrophage cell viability). However, at 60 *μ*M concentrations, FKA was able to suppress macrophage cell viability by ~20% (Figure 7(a)).

Later, the effect of time on the FKA-mediated antioxidant protein expression in RAW 264.7 cells was determined. Cells were treated with 30 *μ*M of FKA for 1-24 h. Subsequently, the expression pattern of Nrf2, HO-1, and NQO-1 proteins was measured by the western blot method. As shown in Figure 7(b), FKA has a differential effect on the expression patterns of these proteins at various time points. The expression of Nrf2 and HO-1 proteins were initiated 1 h after the FKA treatment that lasted up to 12 h. However, the substantial expression of the NQO-1 protein occurred from an 8 h time point and increased towards the longer time points (Figure 7(b)). Hence, FKA has a significant effect on the antioxidant protein expression in murine macrophage cells (Figure 7(b)). We further tested the effect of FKA concentration on the LPS-stimulated ROS generation in RAW 264.7 cells. Macrophages were pretreated with different concentrations of FKA (0, 2.5-10 *μ*M) for 2 h followed by stimulated with or without LPS (1 *μ*g/mL) for 24 h. The generation of intracellular ROS levels was measured through the DCF fluorescence method using fluorescence microscopy. Our data showed that in LPS alone-stimulated cells, ROS levels were dramatically increased (Figures 7(c) and 7(d)). However, this effect was suppressed with increasing concentrations of FKA with a significant suppression observed at 10 *μ*M FKA concentration (Figures 7(c) and 7(d)). These results provided direct evidence that FKA plays a key anti-inflammatory and antioxidant role through the induction of Nrf2, HO-1, and NQO-1 antioxidant gene expression in LPS-stimulated macrophages.

## 4. Discussion

Inflammatory responses are a defense mechanism against infection and injury. But, sustained inflammation is a condition caused by the overexpression of several proinflammatory cytokines or their associated derivatives [29]. For example, iNOS and COX-2 are often responsible for chronic inflammation leading to various inflammation-associated diseases including different types of cancers [30]. The proinflammatory transcription factor NF*κ*B is one of the key factors that regulate these cytokine productions. Therefore, modulating proinflammatory mediators in the macrophages is a good strategy for the treatment of various inflammatory diseases. In this connection, flavonoids, with the characteristic feature of inhibiting the induction of inflammatory cytokines are considered to be an interesting tool for the control of inflammation [31]. In this study, using primary splenocytes isolated from the BALB/c mice as the cellular model system, we demonstrated the anti-inflammatory efficacy of FKA. MTT data showed that FKA (Figure 1(a)) treatment has a negligible effect on the splenocyte cell viability. However, 2.5 *μ*g/mL of LPS (used as a positive control) has significantly increased the proliferation of primary splenocytes (Figure 1(b)). Similar observation was also reported in one of the previous reports in which LPS has significantly promoted the proliferation of splenocytes from WT mice. Their results also demonstrated that LPS and CD40L synergistically stimulated proliferation of mouse splenocytes in a TLR4-dependent manner [18]. ELISA data showed that FKA has a differential effect on the secretion of pro- (TNF-*α*, IL-1*β*, and IL-2) and anti-inflammatory cytokines (IL-10). Proinflammatory cytokines were downregulated whereas, the anti-inflammatory cytokine was upregulated which is inferring that FKA exerts anti-inflammatory properties. (Figures 1(c)–1(f)).

LPS-stimulated macrophages are the widely used cellular model in the anti-inflammatory studies [32, 33]. Therefore, in the current study, primary splenocytes were stimulated with LPS to initiate an inflammatory reaction and the effect of FKA was demonstrated. Our data showed that 2.5 *μ*g/mL of LPS-stimulated the dramatic upregulation of TNF-*α*, IL-1*β*, IL-2, and IL-10 cytokines. However, FKA pretreatment has significantly downregulated the secretions of TNF-*α*, IL-1*β*, and IL-2 at all of the concentrations of FKA. Moreover, in the case of IL-10, this suppression was observed at 30 *μ*M FKA only. This data provides us the fact that FKA acts as a potent anti-inflammatory agent that protects the splenocytes from the inflammatory insult (Figure 2).

Recent evidence suggests that ROS is involved in inflammatory reactions, and LPS-induced ROS generation was widely studied in various *in vitro* and *in vivo* systems [34, 35]. Of particular importance to this study, ROS are oxidative products released by the mitochondria, peroxisomes, cytochrome p450 metabolism, and inflammatory cell activation from endotoxins in macrophages [36]. Consistent with the literature reports, our flow cytometry study data also showed that LPS alone treatment has dramatically upregulated the ROS levels in primary splenocytes. However, this effect was significantly and dose dependently suppressed due to FKA pretreatment (Figure 3(a)). Consistent with this, western blot data also indicated that the expression of nuclear p65, iNOS, COX-2, TNF- *α*, and IL-1*β* proteins were significantly upregulated due to the LPS challenge. However, FKA pretreatment has significantly attenuated this effect signifying that FKA blocked the transcriptional activation of p65 leading to the downstream suppression of inflammatory enzymes and cytokine protein expression (Figures 3(b)–3(f)).

Previous studies have reported that plant-derived natural products can mediate chemoprevention and cytoprotection through anti-inflammatory and antioxidant mechanisms (Nrf2/ARE) [37]. A well-known antioxidant enzyme, HO-1, has an important role in the defense of LPS-induced ROS generation and inflammation in macrophages [38]. Evidence from the literature has suggested that HO-1 expression is driven by Nrf2 and the products of this activity likely inhibit NO production [39]. The induction of antioxidant genes is the mechanism (HO-1, NQO-1, and *γ*-GCLC) that allows Nrf2 to inhibit LPS-induced inflammation [40, 41]. Our western blot data showed that FKA enhanced the nuclear accumulation of Nrf2, a transcription factor for antioxidant gene expression, inducing the expression of antioxidant genes HO-1, NQO-1, and *γ*-GCLC (Figure 4). This was in line with previous reports that indicated many plant extracts do contain anti-inflammatory agents and inhibit ROS formation via the activation of Nrf2 cascades in macrophages [14].

The detailed molecular mechanism on FKA-mediated Nrf2 translocation was not yet fully established. We assume that increased basal ROS levels (low concentrations) by FKA within a short time may attribute to activate the Nrf2 in splenocytes. Though ROS is harmful, low concentrations of ROS are speculated to be involved in redox signaling that may contribute to normal cellular functions and in the prevention of diseases [27]. Erlank et al. reported that in the arterioles and capillaries, in association with the outer surface of the cells and by diffusion, low concentrations of polyphenols generate H_2_O_2_ that activates Nrf2 signaling and cell adaptation to oxidative stress [42]. In another study, Nakazato et al. demonstrated that catechin (a polyphenol from green tea)-mediated ROS was involved in the induction of apoptosis in human malignant cells. It was also explained that polyphenol-induced ROS is capable of modifying the SH-residue in Keap-1 that may lead to Nrf2 activation [43]. When ROS induces the oxidation of specific cysteine residues of Keap-1, Nrf2 can no longer sequester by Keap-1 and is subsequently translocated into the cell nucleus, and then binds to the promoter and activates the transcription of antioxidant enzymes [44]. By keeping this in view, in the current study, we tested the effect of FKA on the basal ROS levels in primary splenocytes that are involved in the activation and translocation of Nrf2. Our flow cytometry data showed that splenocytes exposed to FKA exhibited remarkable increase in the basal ROS levels within a short duration of time (5 min) signifying the fact that FKA-induced ROS is critically involved in the activation of the Nrf2/ARE signaling pathway in splenocytes (Figure 5).

Besides primary splenocytes, the anti-inflammatory efficacy of FKA was demonstrated in one of the widely used *in vitro* experimental cellular models, RAW 264.7, a murine macrophage cell line. The fluorescence microscope data demonstrated that LPS-induced tremendous ROS production (~8-fold) in RAW 264.7 cells, which was dramatically diminished in the presence of FKA (Figures 7(c) and 7(d)). Also, FKA promoted the upregulation of the expressions of Nrf2, HO-1, and NQO-1 proteins in these cells (Figure 7(b)). Interestingly, similar to primary splenocytes, FKA did not show a significant deleterious effect on the viability of RAW264.7 macrophages signifying that FKA is safe to use as an anti-inflammatory agent (Figure 7(a)).

Pancreatitis is an inflammatory disorder of the pancreas with symptoms ranging from mild to death. Cholecystokinin (CCK) or, its predominant active form, sulfated carboxy-terminal octapeptide (CCK-8) were reported to be used as inducers of experimental pancreatitis that increases the secretion of immunoglobulins *in vivo* and modulates lymphoproliferation [45, 46]. CCK-8 develops from a large amount of white pulp that acts as a chemoattractant for human monocytes and rat macrophages in the spleen [47]. Elevated plasma CCK concentrations were observed in patients as well as in various animal models of acute pancreatitis [48]. The *in vivo* anti-inflammatory efficacy of FKA was demonstrated in CCK-8-induced experimental pancreatitis in BALB/c mice using serum lipase levels as the marker enzyme. Consistent with the *in vitro* data obtained from primary splenocyte or RAW 264.7 cell lines, *in vivo* data also demonstrated that in the presence of CCK-8 there was a significant upregulation of serum lipase levels inferring the fact that CCK-8-induced strong inflammatory pancreatitis in the mice. However, pretreatment with FKA attenuated the serum lipase levels (Figure 6). Li et al. tested the biochemical serum analysis of various enzymes as well as the histopathological examination of the liver, kidney, colon, lung, heart, spleen, and thymus tissues for the *in vivo* demonstration of FKA-induced toxicity in mice. In that study, the effect of FKA on the functionality of the pancreas was tested by measuring the activity of serum lipase enzyme as a marker enzyme. Results from their study (biochemical serum analysis and histopathological) has confirmed normal organ function in FKA-treated mice inferring the protective effects of FKA at tissue and biochemical levels [49]. Based on this report, in our study, we speculate that the preadministration of FKA might be reversing the malfunctioning of the pancreas (experimentally induced due to cholecystokinin) at the tissue level leading to the reduction in the serum lipase levels when compared to the mice that did not take FKA.

This was also evidenced by the expression profiles of various pro- and anti-inflammatory cytokines secreted from the control-, LPS- or concanavalin A-stimulated primary splenocytes derived from the FKA-pretreated BALB/c mice. Con A is a plant mitogen used to study the immune regulation of various immune cells because it can stimulate mouse T cell subsets and shows four functionally distinct T cell populations [50]. Our data demonstrated that due to the FKA pretreatment, control -, LPS- and Con A-stimulated primary splenocytes showed a significant downregulation of TNF-*α*, IL-1*β*, IL-2, and IL-6 proinflammatory cytokines. Interestingly, control, LPS, or Con A has no significant effect on IL-10 secretions (Table 1) suggesting that FKA is a potent anti-inflammatory and antioxidant Kava-kava derivative that has beneficial effects in the development of novel therapeutic compounds to treat diseases associated with the dysregulation of inflammatory cells.

## 5. Conclusion

In summary, the sublethal concentrations of chalcone FKA inhibited ROS, NF*κ*B, iNOS, and COX-2 expressions leading to the suppression of proinflammatory TNF-*α*, IL-1*β*, IL-2, and IL-6 secretions in the LPS-stimulated primary splenocytes. We demonstrated that the anti-inflammatory and antioxidant efficacy of FKA is leading to the induction of antioxidant genes HO-1, NQO-1, and *γ*-GCLC via the Nrf2/ARE signaling pathways. The *in vivo* study results also confirmed that FKA pretreatment has an important role in the downregulation of serum lipase levels in the CCK-8-induced experimental pancreatitis in BALB/c mice. Therefore, chalcone flavokawain A could be a promising plant-derived immunomodulatory and antioxidant agent that can be used to treat the diseases associated with inflammatory disorders.

## Figures and Tables

**Table 1:** Pro- and anti-inflammatory cytokine secretion patterns in saline-, LPS-, or Con A-stimulated primary splenocytes derived from FKA pretreated BALB/c mice. LPS- or Con A-stimulated TNF-*α*, IL-1*β*, IL-2, and IL-6 secretions were suppressed in primary splenocytes derived from the FKA-pretreated BALB/c mice. BALB/c mice were administered with 30 mg/kg of FKA for 4 h. After the treatment, primary splenocytes were isolated and maintained. These cells were stimulated with saline, LPS (2.5 *μ*g/kg), or Con A (2.5 *μ*g/kg) for 72 h, and the secretions of proinflammatory TNF-*α*, IL-1*β*, IL-2, and IL-6 cytokines as well as anti-inflammatory IL-10 cytokine were measured using the commercial ELISA kits. Results were represented as the mean ± SD of three experiments. Statistical significance was assigned as ^∗^*p* < 0.05; ^∗∗^*p* < 0.01; and ^∗∗∗^*p* < 0.001 compared to the values obtained from primary splenocytes that were derived from the mice not administered with FKA.

Cytokine (pg/mL)	Treatment	Control (*n* = 4)	FKA (30 mg/kg) (*n* = 4)
TNF-*α*	Saline	110.0 ± 21.6	33.5 ± 6.5^∗^
	LPS	250.7 ± 23.3	40.0 ± 7.3^∗∗∗^
	Con A	194.4 ± 30.8	84.5 ± 19.6^∗^

IL-1*β*	Saline	25.9 ± 0.6	18.5 ± 1.3^∗∗^
	LPS	46.2 ± 5.0	25.4 ± 0.9^∗∗^
	Con A	21.3 ± 1.6	18.0 ± 0.9

IL-2	Saline	16.2 ± 2.3	7.1 ± 2.0^∗^
	LPS	19.2 ± 4.2	9.2 ± 2.1
	Con A	885.7 ± 187.8	632.1 ± 229.6

IL-6	Saline	8.5 ± 0.1	6.9 ± 0.5^∗∗^
	LPS	58.6 ± 6.1	7.9 ± 1.7^∗∗∗^
	Con A	30.9 ± 2.7	17.5 ± 4.6^∗^

IL-10	Saline	20.8 ± 0.2	21.8 ± 1.1
	LPS	117.7 ± 8.6	101.9 ± 20.1
	Con A	59.8 ± 21.3	65.3 ± 22.2

TNF-*α*/IL-10	Saline	5.194 ± 0.883	1.543 ± 0.298^∗^
	LPS	2.605 ± 0.206	0.425 ± 0.105^∗∗∗^
	Con A	3.503 ± 0.571	1.790 ± 0.343

IL-6/IL-10	Saline	0.409 ± 0.011	0.317 ± 0.014^∗∗∗^
	LPS	0.522 ± 0.081	0.078 ± 0.105^∗∗^
	Con A	0.778 ± 0.201	0.308 ± 0.019

(a) Values were shown as mean ± SD. (b) ^∗^*p* < 0.05, ^∗∗^*p* < 0.01, and ^∗∗∗^*p* < 0.001 represents significantly different from the ‘control'. (c) The sensitivity limitation of ELISA kits used in this study was <15.6 pg/mL. (d) The treatment concentrations of lipopolysaccharide (LPS) and concanavalin A (Con A) were 2.5 *μ*g/mL.

## Data Availability

The data used to support the findings of this study are included within the article.

## Abbreviations

TNF-*α*: Tumor necrosis factor-*α*
IL-1*β*: Interleukin-1*β*
IL-2: Interleukin-2
IL-10: Interleukin-10
LPS: Lipopolysaccharide
iNOS: Inducible nitric oxidase
COX-2: Cyclooxygenase-2
HO-1: Hemoxygenase-1
NQO-1: NADPH dehydrogenase-1
*γ*-GCLC: Glutamate-cysteine ligase catalytic subunit
CCK-8: Cholecystokinin-8
Con A: Concanavalin A.
